# The Association between Remnant Cholesterol Levels and Computed Tomography-based Low Bone Mass, Low Muscle Mass, and Osteosarcopenia in Older Adults

**DOI:** 10.2174/0115734056432895260322132111

**Published:** 2026-04-03

**Authors:** Rongzhou Wang, Yu Wang, Jingjing Liu, Zicheng Wei, Hongye Tang, Xiao Chen

**Affiliations:** 1 Department of Radiology, Affiliated Hospital of Nanjing University of Chinese Medicine, Nanjing, China

**Keywords:** Serum lipids, Remnant cholesterol, Low bone mass, Sarcopenia, Osteosarcopenia, Muscle quality

## Abstract

**Objective:**

The associations between remnant cholesterol (RC) and low bone mass and sarcopenia have not been well studied. This study aimed to demonstrate the link between serum RC and the prevalence of low bone mass, low muscle mass, and osteosarcopenia in older adults.

**Methods:**

This retrospective study involved 1995 individuals aged 50 and above who received CT scans for lung cancer screening from 2016 to 2020. Remnant cholesterol (RC) was calculated as follows: total cholesterol (TC) - (low-density lipoprotein-cholesterol (LDL-c) + high-density lipoprotein-cholesterol (HDL-c)). Low bone mass was defined as bone CT attenuation < 110 HU. Low muscle mass was defined when the area of the erector spinae muscles was < 20 cm^2^ in women and < 25 cm^2^ in men. Osteosarcopenia was defined as bone CT attenuation < 110 HU and muscle area < 20 cm^2^ in women or < 25 cm^2^ in men.

**Results:**

A total of 469 (23.5%) patients with low bone mass, including 268 women and 201 men, were observed. Serum RC was not associated with low bone mass. Elevated serum RC was associated with a lower prevalence of low muscle mass in both women and men (OR = 0.71, 95% CI: 0.50-1.00; OR = 0.53, 95% CI: 0.38-0.75). Serum RC was also associated with osteosarcopenia in women (OR = 0.59, 95% CI: 0.34-1.00) and men (OR = 0.53, 95% CI: 0.29-0.99).

**Discussion:**

RC may be a valuable factor for the early identification and management of sarcopenia or osteosarcopenia. However, further studies are needed to investigate the causal relationships and the mechanisms by which RC affects bone and muscle quality.

**Conclusion:**

Serum RC was not associated with low bone mass but was negatively associated with low muscle mass and osteosarcopenia in older adults.

## 1 INTRODUCTION

Osteoporosis and dyslipidemia are both major global health concerns, particularly common among older adults [1, 2]. The relationship between blood lipid levels-including total cholesterol (TC), triglycerides (TG), low-density lipoprotein cholesterol (LDL-c), and high-density lipoprotein cholesterol (HDL-c)-and bone mineral density (BMD) or bone metabolism has been extensively studied. HDL-c, often referred to as “good” cholesterol, has been positively associated with BMD in some studies [3, 4], while elevated levels of LDL-c and TG have been linked to reduced bone density [5, 6]. However, findings remain inconsistent. For instance, some studies have reported an inverse association between HDL-c and BMD [7, 8], and a positive correlation between LDL-c and BMD in postmenopausal women [8]. These conflicting results suggest that the relationship between lipid profiles and bone health warrants further investigation.

Sarcopenia, another age-related condition, is characterized by progressive loss of muscle mass and strength, leading to reduced mobility, increased risk of falls and fractures, and diminished quality of life [9]. Emerging evidence suggests a potential link between dyslipidemia and muscle loss. Jiang *et al*. found that low levels of TC and TG, along with high LDL-c, were associated with sarcopenia [10]. Conversely, other studies have reported inverse associations between TC, TG, LDL-c, and skeletal muscle index [11-13], while a positive association between HDL-c and sarcopenia has also been observed [12-14]. Interestingly, a high TG/HDL-c ratio, indicating either elevated TG or low HDL-c, has been linked to a reduced risk of severe sarcopenia in some studies [12, 15, 16]. These contradictory findings highlight the need for further research to clarify the role of lipid profiles in sarcopenia development.

Remnant cholesterol (RC), also known as triglyceride-rich lipoprotein cholesterol, refers to cholesterol not contained within HDL-c or LDL-c, including very low-density lipoproteins (VLDL) and intermediate-density lipoproteins (IDL) [17]. RC has been implicated in cardiovascular [18] and metabolic diseases [19]. Recent studies also suggest a link between RC or the RC/HDL-c ratio and bone loss in younger and middle-aged populations [20-22] or in patients with Duchenne muscular dystrophy [23]. However, few studies have focused on such associations in the general older population. Similarly, two studies have reported a significant correlation between VLDL and remnant-like particle cholesterol (RLP-C) levels or RC and skeletal muscle index and sarcopenia [11, 24, 25].

In recent years, osteosarcopenia, a unique condition defined by the coexistence of osteopenia or osteoporosis and sarcopenia, has been introduced [24]. A meta-analysis showed that the prevalence of osteosarcopenia was 18.5% [26]. Old age, sex, fragility, and nutritional status are the main factors associated with osteosarcopenia. Osteosarcopenia is related to a higher risk of falls, fractures, hospitalizations, and death than low bone mass or sarcopenia alone [26]. Interestingly, recent studies also revealed that TC and HDL-c were associated with osteosarcopenia [27, 28].

However, to our knowledge, the associations between RC and low bone mass and sarcopenia have not been well studied, and no study has reported the association between remnant cholesterol and osteosarcopenia in older adults. Understanding such associations is valuable for the identification, management, and intervention of musculoskeletal diseases. Muscle mass is an essential element in the conceptual definition of sarcopenia [29]. Bone mass and muscle mass are usually measured by Dual-energy X-ray Absorptiometry. Computed tomography (CT) has also been used to assess bone mass and muscle mass [30, 31]. In this investigation, we analyzed the associations between RC and CT-based prevalence of low bone mass, low muscle mass, and osteosarcopenia in a Chinese population aged 50 years or older. Our research will elucidate the relationship between blood lipids and osteoporosis, sarcopenia, and osteosarcopenia from a novel perspective.

## 2 METHODS

### 2.1 Participants

Opportunistic osteoporosis screening on chest CT, originally performed for lung cancer detection, has already been validated [32, 33]. The approach adds neither extra cost nor additional ionizing radiation. Leveraging this advantage, we conducted a retrospective analysis to examine whether RC is associated with CT-defined low bone mass, low muscle mass, and osteosarcopenia. Inclusion criteria: aged 50 years and above; had CT examinations for lung cancer detection in our institution from 2016 to 2020. The exclusion criteria were as follows: 1) history of tumors or cancer, stroke, or severe heart disease (heart failure or coronary heart disease); 2) diseases that may affect bone health, such as autoimmune or rheumatic diseases, parathyroid dysfunction, and osteomalacia, or use of glucocorticoids for more than three months; 3) unavailable data on serum lipids; 4) severe kidney or liver dysfunction; and 5) absence of spine CT images. Finally, 1995 subjects were included for analysis. This study was approved by the Ethics Committee of our institution (2019NL-158). After application of these criteria, 1,995 participants remained for analysis. The study protocol was approved by the Institutional Ethics Committee (2019-NL-158) and followed the Declaration of Helsinki. Because of its retrospective nature, the Ethics Committee waived the requirement for informed consent.

### 2.2 Data Collection

Clinical and laboratory data-including age, sex, medical history, body-mass index (BMI), laboratory test results (TG, TC, LDL-c and HDL-c, aspartate aminotransferase (AST), renal function biomarkers (creatinine), levels of blood glucose, serum albumin, and alkaline phosphate (ALP) were obtained from electronic medical records. Albumin served as a marker of nutritional status. Diabetes was identified from either prior diagnoses or fasting glucose levels. The RCs were cholesterol components calculated via the following equation: TC - (HDL-c +LDL-c) [34]. Categorical RC data (divided by the median of the overall population) were also included in the statistical analysis.

### 2.3 Bone and Muscle Measurement

**Fig. (1):**
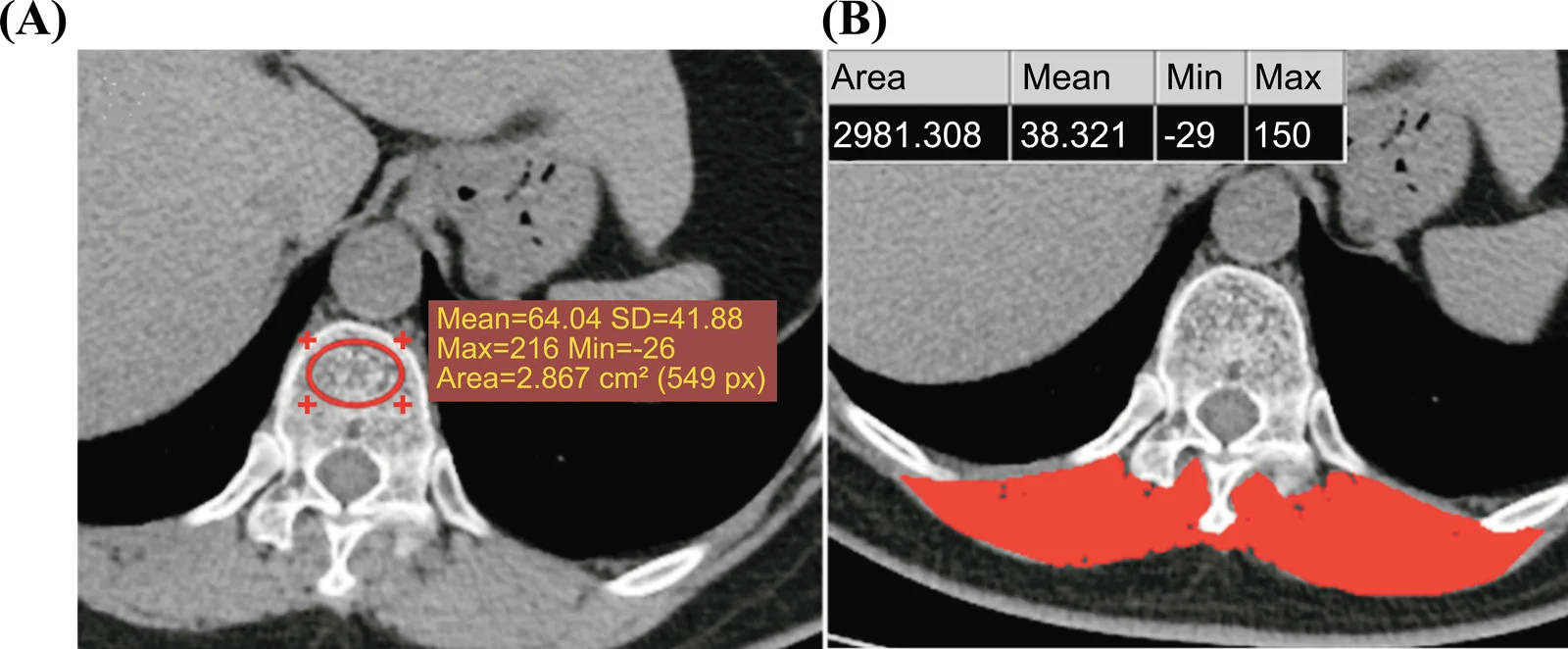
The illustration for measurements of bone attenuation (**A**), muscle area, and attenuation (**B**). The bone computed tomography (CT) attenuation and the area and attenuation of the erector spinae muscles were measured on axial CT images at the mid-level of the T11 vertebrae. The regions of interest were drawn using ImageJ software (Version 1.54, USA).

Bone mass, muscle area, and muscle density were all derived from the same chest CT dataset. Because spine BMD was not available, we used trabecular CT attenuation as a surrogate; this metric correlates strongly with dual-energy X-ray BMD [35]. The detailed information on these measurements has been reported in previous studies [36, 37]. Acquisition parameters were 120 kV, 100-130 mAs, and 0.625 mm slice thickness. On a single axial slice through the mid-T11 vertebral body, a circular 20-30 mm^2^ ROI was placed in the trabecular compartment to record mean Hounsfield units (HU). At the same level, the bilateral erector spinae muscles were segmented with ImageJ to obtain total cross-sectional area (cm^2^) and mean density (HU). Figure **1** illustrates the ROI placement for both measurements.

### 2.4 Low Bone/muscle Mass and Osteosarcopenia Definitions

Low bone mass was defined as trabecular CT attenuation < 110 HU [38]. Sex-specific cut-offs for muscle parameters were set at the first quartile observed in our cohort. Consequently, low muscle area was < 20 cm^2^ in women and < 25 cm^2^ in men; low muscle density was < 32 HU in women and < 38 HU in men. Osteosarcopenia was present when both low bone mass (trabecular attenuation < 110 HU) and low muscle area criteria were fulfilled. The study protocol is shown in Fig. (**2**).

### 2.5 Statistical Analysis

Quantitative variables are presented as mean ± SD and categorical variables as counts. Group differences were evaluated with independent-sample t tests or Mann-Whitney U tests, as appropriate. Because sex-specific cut-offs were used for low muscle mass and density, all analyses were stratified by sex. Multivariable logistic regression was employed to estimate the association between serum remnant cholesterol (RC) and each bone or muscle outcome. Models were adjusted for age, BMI, diabetes, serum albumin, and AST; albumin and AST were included to account for nutritional status and potential hepatic effects on bone and muscle [39, 40]. RC was analyzed both as a continuous variable and as a binary variable split at the cohort median. Statistical analyses were conducted via SPSS version 20.0. Sample size was estimated using PASS11.0 software: α = 0.05, power = 0.9, P0 = 5%, odds ratio (OR) = 0.7, R square = 0.1. The estimated sample size was 1932. A *P*-value < 0.05 was considered to indicate statistical significance.

## 3 RESULTS

### 3.1 Characteristics of the Subjects

The cohort comprised 837 women and 1,158 men (Table **1**). Mean age was 62.39 ± 9.53 years overall: 61.91 years for women and 62.74 years for men. The prevalence of low bone mass, low muscle mass, and osteosarcopenia was 23.5%, 27.8%, and 11.6%, respectively. Women exhibited lower trabecular bone attenuation (*P* < 0.01), smaller muscle area, and lower muscle density (both *P* < 0.01) than men. Serum albumin, ALP, TC, RC, HDL-C, and LDL-C levels were all significantly higher in women (*P* < 0.01).

### 3.2 Associations between Serum RC levels and low Muscle/bone Mass

After adjustment for age, BMI, and diabetes mellitus, serum RC was inversely associated with low muscle mass in both sexes (women: OR = 0.44, 95% CI 0.51-0.99; men: OR = 0.55, 95% CI 0.39-0.77) (Table **2**). The association persisted in the fully adjusted model (further controlling for albumin and AST): women OR = 0.71 (95% CI 0.50-1.00), men OR = 0.53 (95% CI 0.38-75). When RC was analyzed categorically (cut-off 0.32 mmol/L), high RC remained protective against low muscle mass in both women (OR = 0.70, 95% CI 0.50-0.97) and men (OR = 0.71, 95% CI 0.53-0.94) under full adjustment. In contrast, serum RC was not independently associated with low muscle density (Table **3**) or low bone mass (Table **4**) in either sex (all *P* > 0.05), and similar null findings were observed in the overall cohort. The illustrations of low muscle mass in subjects with low or high levels of RC are shown in Fig. (**3**).

### 3.3 Association between Serum RC level and Osteosarcopenia

An association between serum RC and osteosarcopenia was observed in men (OR = 0.54, 95% CI: 0.30-0.99) (Table **5**). In the fully adjusted model, which was additionally adjusted for serum ALB and AST, this factor was still associated with osteosarcopenia (OR = 0.53, 95% CI 0.29-0.99) (Table **5**). For women, the level of serum RC was also independently associated with osteosarcopenia after additional adjustment for confounding factors (OR = 0.59, 95% CI 0.34-1.00). High serum RC levels (> 0.32 mmol/L) were associated with a risk of low muscle mass in men (OR = 0.38, 95% CI:0.15-0.95) in the fully adjusted model, but not in women (OR = 0.62, 95% CI:0.38-1.01). For the overall population, both continuous and categorical data of RC were associated with osteosarcopenia (OR = 0.50, 95% CI:0.31-0.80; OR = 0.55, 95%CI:0.36-0.83).

## 4 DISCUSSION

The relationships between conventional lipid fractions-LDL-C and HDL-C-and the risks of osteoporosis [3-8] and sarcopenia [11-16] have been extensively examined. More recently, remnant cholesterol (RC) has emerged as a novel lipid marker linked to diverse health outcomes [18, 19], yet its connection with BMD, sarcopenia, or osteosarcopenia remains largely unexplored. In the present study, higher serum RC was independently associated with a lower probability of low muscle mass in both sexes after full adjustment. Additionally, elevated RC was related to a reduced likelihood of osteosarcopenia in men, but this association was not observed in women.

To our knowledge, only two studies have explored the link between serum RC and BMD. Hou *et al*. found an inverse correlation between RC and hip BMD in Chinese men [20], and a U.S. cohort reported a similar association with total-spine BMD [21]. We did not replicate these findings in our older population. The discrepancy is likely explained by age differences: the earlier cohorts were young to middle-aged (mean 43 and 39 years), whereas our participants were all ≥ 60 years. In addition, the RC levels in our study (75^th^ percentile: 0.61 mmol/L) seemed lower than those reported in published studies (75^th^ percentile: 0.70 mmol/L and 0.90 mmol/L). The association between RC and BMD was not observed in Xiao’s study when the RC level was < 0.7 mmol/L [21] or in Hou’s study when the RC level was < 0.9 mmol/L [20]. Moreover, the change in hip or spine BMD with increasing RC was very small (β = - 0.0079 or β = -0.024), even though it was statistically significant. RC levels may have no potential value in osteoporosis management in older adults.

Although dyslipidaemia and sarcopenia have been linked in several studies, direct evidence on remnant cholesterol (RC) and muscle mass is scarce. Gong *et al*. [11] was the only group to date to report an inverse correlation between RLP-C-a constituent of RC-and skeletal muscle index (SMI). Our findings are in contrast: higher RC was associated with a lower risk of low muscle area. The discrepancy may reflect methodological differences. Gong’s cohort was small (n = 84) and older (mean age 75 years), whereas our sample is larger and spans an earlier segment of older adulthood. Notably, the same authors also observed a positive association between VLDL (another RC component) and SMI [11], a result that aligns with our data. Taken together, these observations suggest that RC could offer clinically relevant information for sarcopenia or osteosarcopenia management in older adults.

Muscle area was positively correlated with muscle density in our study (data not shown). However, we did not know why RC was not associated with muscle density. When we measured the muscle CT attenuation, the ROIs were placed on the muscle, avoiding areas with obvious visible fat infiltration. The muscle density may be greater than that measured in the whole muscle. Another possible explanation was that age may play a different role in the associations between RC and muscle mass and muscle density.

Osteosarcopenia-simultaneous low bone and low muscle mass-has emerged as a distinct geriatric syndrome [24]. Data on its relationship with serum lipids, and especially with remnant cholesterol (RC), are sparse. One recent report showed that higher total cholesterol (TC) reduced the odds of osteosarcopenia [27]; because TC and RC track together, we hypothesised that RC might confer similar protection. Indeed, elevated RC was linked to a 41-47% lower risk in our cohort (men: OR 0.59, 95% CI 0.34-1.00; women: OR 0.53, 95% CI 0.29-0.99). The underlying mechanisms await investigation.

There were several innovations in our study. First, although a link between RC or the RC/HDL-c ratio and bone loss has been reported [20-23], few studies have focused on such associations in the general older population. Second, currently, only a handful of studies have examined the relationship between RC and sarcopenia, yet very few have analyzed its association with muscle density. Third, to our knowledge, no study has reported the association between RC and osteosarcopenia in older adults.

## 5 STUDY LIMITATIONS

Our study has several limitations. First, we did not explore potential biological mechanisms, as the pathways through which RC may influence bone and muscle remain unclear. Second, low bone mass, low muscle mass, and osteosarcopenia were defined by CT rather than by established guidelines. Although CT-derived trabecular attenuation and muscle area are widely accepted surrogates [32, 41-43], our findings need validation with dual-energy X-ray absorptiometry or bioelectrical impedance analysis. Third, we lacked bone turnover markers and muscle-specific biochemical indices that could have refined phenotype characterization. Fourth, despite adjustment for multiple covariates, data on diet, physical activity, and visceral adiposity were unavailable. Fifth, sarcopenic obesity was rare in our cohort (12 women, 19 men), precluding meaningful statistical analysis. Finally, the cross-sectional design precludes causal inference; therefore, longitudinal studies are required.

## CONCLUSION

In summary, this study revealed associations between serum RC levels and the risk of low bone mass, low muscle mass, and osteosarcopenia in the older population. Previous studies have shown varying associations of RC with bone mass in different populations. Our results demonstrated that serum RC levels were not associated with low bone mass but were negatively associated with the prevalence of low muscle mass and osteosarcopenia in both women and men. Our study reports new lipid-related indicators associated with sarcopenia, low muscle mass, and osteosarcopenia. RC may be a valuable factor for the early identification and management of sarcopenia or osteosarcopenia. However, further studies are needed to investigate the causal relationships and the mechanisms by which RC affects bone and muscle quality.

## Figures and Tables

**Fig. (2):**
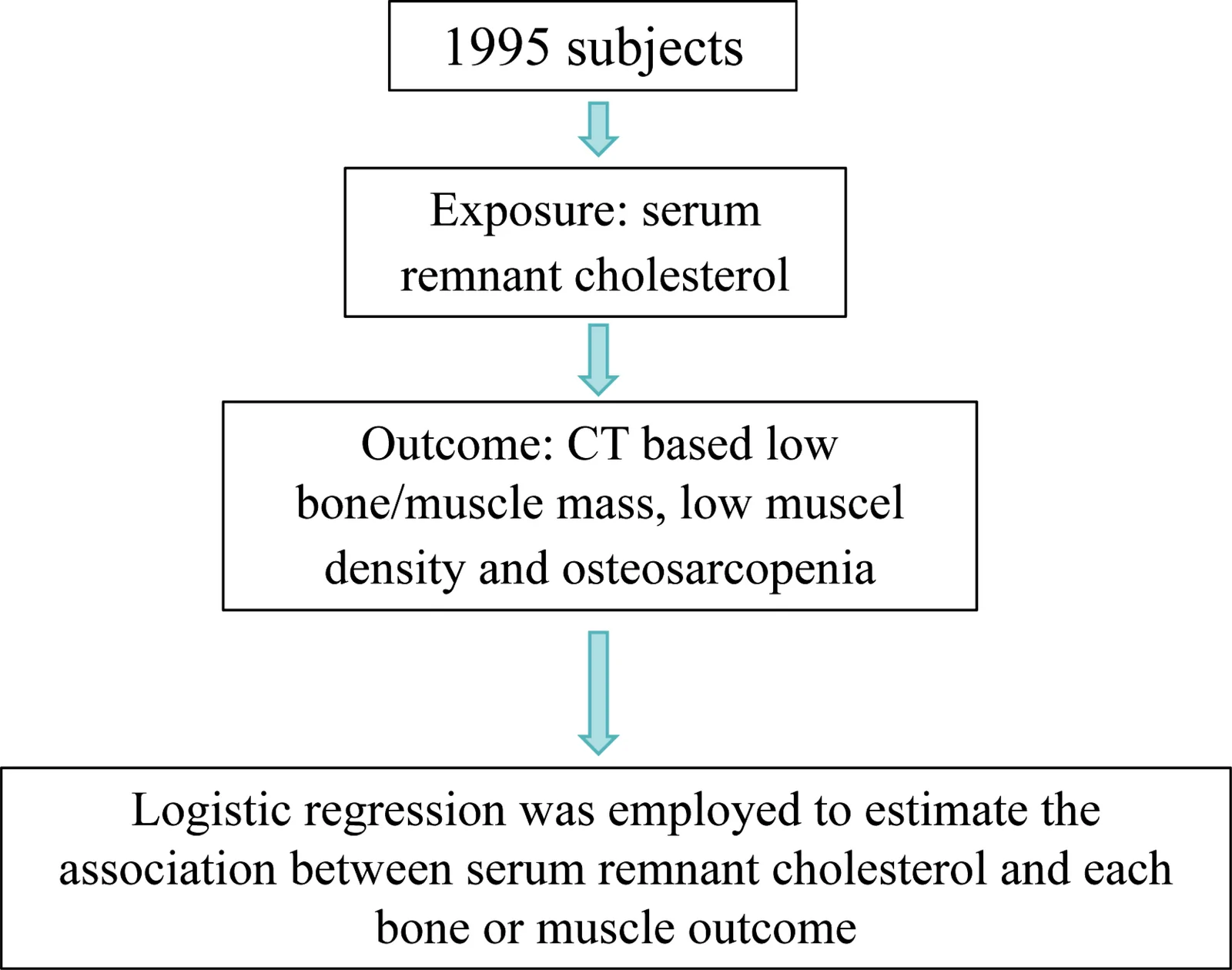
The study protocol.

**Fig. (3):**
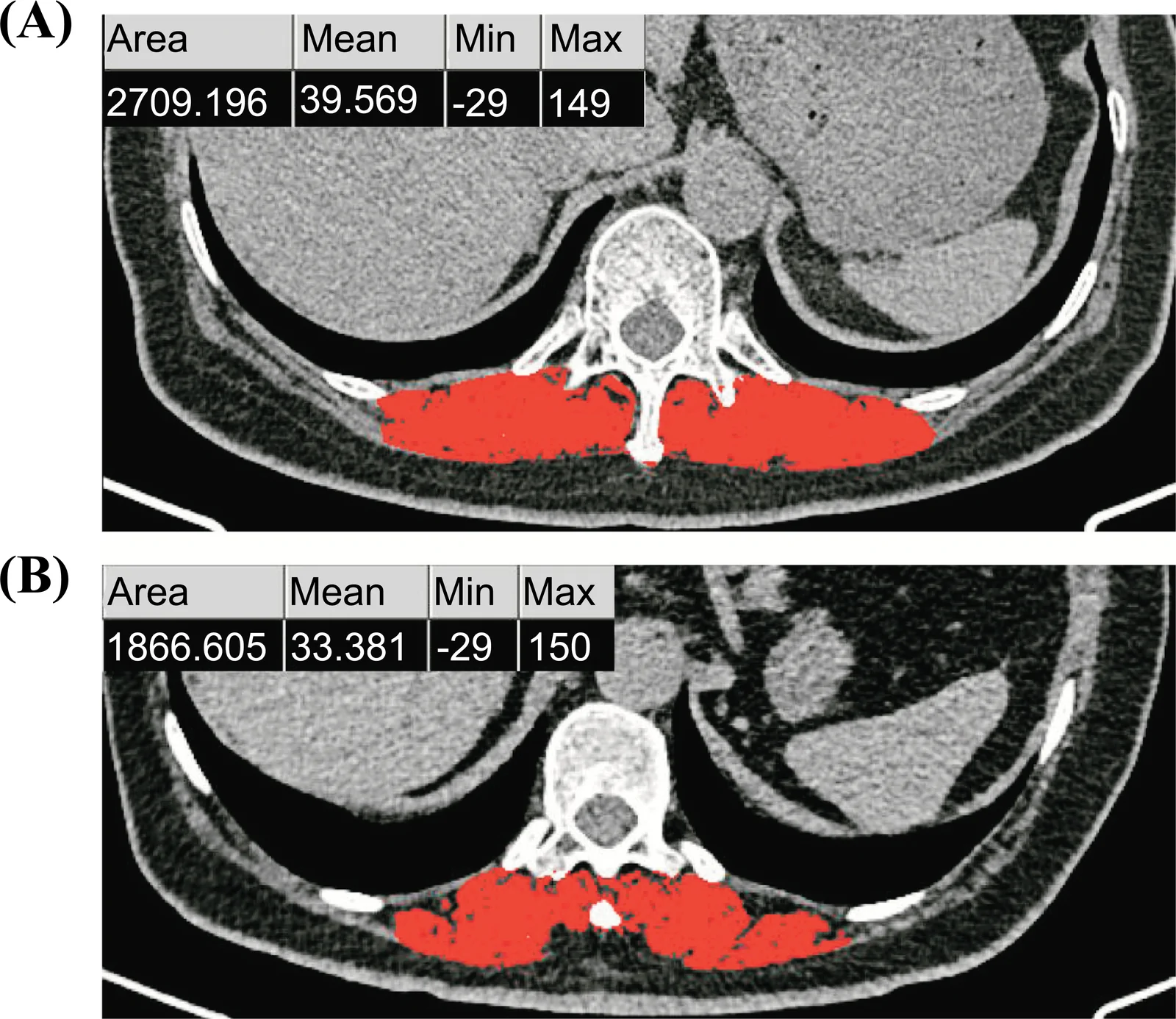
The illustration of low muscle mass in subjects with low or high levels of remnant cholesterol (RC). A 71 years old women with 0.36 mmol/L of RC had a high muscle mass (27.1 cm^2^) (**A**); A72 years old women with 0.13 mmol/L of RC had a low muscle mass (18.7 cm^2^) (**B**).

**Table 1:** The characteristics of subjects.

	Women (n = 837)	Men (n = 1158)	P
Age (years)	61.91 ± 9.48	62.74 ± 9.56	0.06
BMI (kg/m^2^)	25.41 ± 2.03	23.85 ± 1.91	< 0.001
Bone CT attenuation (HU)	136.72 ± 48.06	145.55 ± 38.60	< 0.001
Low bone mass (n)	268	201	< 0.001
AST (mmol/L)	24.00 ± 13.86	24.06 ± 7.73	0.89
Albumin (mmol/L)	41.08 ± 3.09	40.53 ±3.12	< 0.001
ALP (mmol/L)	80.97± 21.68	75.81 ± 21.88	< 0.001
Creatinine (mmol/L)	66.29 ± 14.07	85.93 ± 25.82	<0.001
Blood glucose (mmol/L)	5.43 ± 1.36	5.64 ± 1.41	0.001
HDL cholesterol (mmol/L)	1.65 ± 0.35	1.46 ± 0.35	< 0.001
LDL-cholesterol (mmol/L)	2.93 ± 0.34	1.39 ± 0.0.31	< 0.001
RC (mmol/L)	0.38 ± 0.34	0.30 ± 0.29	0.001
TC (mmol/L)	5.12 ± 1.02	4.55 ± 0.96	< 0.001
TG (mmol/L)	1.50 ± 1.05	1.57 ± 1.17	0.15
Muscle area (cm^2^)	23.42 ± 5.45	29.45 ± 6.76	< 0.001
Low muscle area (n)	229 (27.4%)	325 (28.1%)	/
Muscle density (HU)	36.74 ± 7.22	42.26 ± 7.25	< 0.001
Low muscle density (n)	202 (24.1%)	271 (32.35)	/

**Abbreviations:** AST: Aspartate aminotransferase; ALP: Alkaline Phosphatase; CT: computed tomography; HDL-c: high-density lipoprotein cholesterol; HU: hounsfield unit; LDL-c: low-density lipoprotein cholesterol; RC: remnant cholesterol; TC: total cholesterol; TG: triglyceride;

**Table 2:** The association between remnant cholesterol and risk of low muscle mass.

	Women				Men			
	Model 1	P	Model 2	P	Model 1	p	Model 2	P
	OR (95%CI)		OR (95%CI)		OR (95%CI)		OR (95%CI)	
RC (continuous data)	0.44 (0.51-0.99)	0.044	0.71 (0.50-1.00)	0.050	0.55 (0.39-0.77)	< 0.001	0.53 (0.38- 0.75)	< 0.001
RC (categorical data)								0.001
Low (< 0.32)	1		1		1		1	
High (> 0.32)	0.70 (0.50-0.97)	0.033	0.70 (0.50-0.98)	0.036	0.72 (0.54-0.96)	0.024	0.71 (0.53-0.94)	0.019

**Notes:** Model 1 was adjusted for age, body mass index, and diabetes. Model 2 was additionally adjusted with serum albumin, creatinine, and aspartate aminotransferase.

**Abbreviations:** CI, confidence interval; OR: odds ratio; RC: remnant cholesterol.

**Table 3:** The association between remnant cholesterol and risk of low muscle density.

	Women (32 HU)				Men (38 HU)			
	Model 1	P	Model 2	P	Model 1	P	Model 2	P
	OR (95%CI)		OR (95%CI)		OR (95%CI)		OR (95%CI)	
RC (continuous data)	0.92 (0.63-1.33)	0.64	0.85 (0.58- 1.25)	0.41	0.86 (0.59-1.28)	0.46	0.86 (0.58- 1.28)	0.47
RC (categorical data)								
Low (< 0.32)	1				1			
High (> 0.32)	1.03 (0.70-1.52)	0.88	1.00 (0.68-1.47)	0.98	1.06 (0.75-1.51)	0.73	1.06 (0.74-1.51)	0.76

**Notes:** Model 1 was adjusted for age, body mass index, and diabetes. Model 2 was additionally adjusted with serum albumin, creatinine, and aspartate aminotransferase.

**Abbreviations:** CI, confidence interval; OR: odds ratio; RC: remnant cholesterol.

**Table 4:** The association between remnant cholesterol and risk of low bone mass.

	Women				Men			
	Model 1	P	Model 2	P	Model 1	P	Model 2	P
	OR (95%CI)		OR (95%CI)		OR (95%CI)		OR (95%CI)	
RC (continuous data)	0.89 (0.66-1.19)	0.43	0.91 (0.67-1.23)	0.53	1.08 (0.77-1.53	0.66	1.05 (0.74- 1.50)	0.77
RC (categorical data)								
Low (< 0.32)	1		1		1			
High (> 0.32)	0.84 (0.62-1.15)	0.28	0.85 (0.63-1.17)	0.32	1.18 (0.87-1.63)	0.29	1.16 (0.85-1.60)	0.35

**Notes:** Model 1 was adjusted for age, body mass index, and diabetes; Model 2 was additionally adjusted with serum albumin, creatinine, and aspartate aminotransferase.

**Abbreviations:** CI, confidence interval; OR: odds ratio; RC: remnant cholesterol.

**Table 5:** The association between remnant cholesterol and risk of osteosarcopenia.

	Women				Men			
	Model 1	P	Model 2	P	Model 1	P	Model 2	P
	OR (95%CI)		OR (95%CI)		OR (95%CI)		OR (95%CI)	
RC (mmol/L)	0.68 (0.40-1.12)	0.16	0.59 (0.34-1.00)	0.050	0.54 (0.30-0.99)	0.048	0.53 (0.29- 0.99)	0.045
HDL(mmol/L)	2.07 (0.93-4.62)	0.076	2.02 (0.91-4.52)	0.085	1.58 (0.69-3.59)	0.28	1.70 (0.74-3.88)	0.21
LDL(mmol/L)	1.03 (0.76-1.40)	0.83	0.99 (0.73-1.35)	0.95	0.96 (0.70-1.32)	0.82	0.96 (0.69-1.32)	0.78
TC(mmol/L)	1.00 (0.80-1.26)	0.98	0.96 (0.76-1.21)	0.72	0.92 (0.72-1.17)	0.48	0.92 (0.72-1.17)	0.49
TG(mmol/L)	0.79 (0.53-1.07)	0.12	0.69 (0.48-1.01)	0.055	0.72 (0.48-1.07)	0.099	0.72 (0.48-1.08)	0.12

**Notes:** Model 1 was adjusted for age, body mass index, and DM; Model 2 was additionally adjusted with serum albumin, creatinine, and aspartate aminotransferase.

**Abbreviations:** CI, confidence interval; OR: odds ratio; RC: remnant cholesterol.

## Data Availability

The data of current study are available from corresponding author, [X.C], on a reasonable request.

## LIST OF ABBREVIATIONS

BMD: Bone Mineral Density
CI: Confidence Interval
CT: Computed Tomography
LDL-c: Low Density Lipoprotein Cholesterol
HDL-c: High Density Lipoprotein Cholesterol
OR: Odds Ratio
RC: Remnant Cholesterol
TC: Total Cholesterol
TG: Triglyceride

## STANDARDS OF REPORTING

STROBE guidelines were followed.
